# Improving malaria preventive practices and pregnancy outcomes through a health education intervention: A randomized controlled trial

**DOI:** 10.1186/s12936-021-03586-5

**Published:** 2021-01-21

**Authors:** Ahmed Dahiru Balami, Salmiah Md. Said, Nor Afiah Mohd Zulkefli, Bachok Norsa’adah, Bala Audu

**Affiliations:** 1 Department of Community Health, Faculty of Medicine and Health Sciences, Universiti Putra Malaysia, Selangor, Malaysia; 2 Unit of Biostatistics and Research Methodology, School of Medical Sciences, Universiti Sains Malaysia, Kelantan, Malaysia; 3 Department of Obstetrics and Gynaecology, University of Maiduguri, Maiduguri, Nigeria

**Keywords:** Randomized controlled trial, Health education, Insecticide-treated net, Intermittent preventive treatment, Birth weight

## Abstract

**Background:**

The prevalence of malaria in pregnancy and its complications, remain very high in Nigeria. This study aimed to determine the effects of a malaria health educational intervention based on the information-motivation-behavioural skills (IMB) model on malaria preventive practices and pregnancy outcomes.

**Methods:**

The study was a randomized controlled parallel-group study, where 372 randomly selected antenatal care attendees were randomly assigned to one of either two groups after collecting baseline data. The intervention group then received a four-hour health education intervention in Hausa language, which was developed based on the IMB model, while the control group received a similarly designed health education on breastfeeding. Follow up data were then collected from the participants at a first (2 months post-intervention) and second (4 months post-intervention) follow up, and at the end of their pregnancies.

**Results:**

For both groups, reported ITN use had increased from baseline (Intervention: Often–14.0%, Almost always–9.1; Control: Often–12.4%; Almost always 16.1%) to the time of second follow up (Intervention: Often –28.10%, Almost always–24.5; Control: Often–17.2%; Almost always 19.5%). Reported IPTp uptake at second follow up was also higher for the intervention group (Intervention: Two doses–59.0%, Three doses 22.3%; Control group: Two doses–48.4%, Three doses–7.0%). The drop in the haematocrit levels was greater for the control group (32.42% to 30.63%) compared to the intervention group (33.09% to 31.93%). The Generalized Linear Mixed Models (GLMM) analysis revealed that the intervention had significantly improved reported ITN use, reported IPTp uptake, and haematocrit levels, but had no significant effect on the incidence of reported malaria diagnosis or babies’ birth weights.

**Conclusions:**

The intervention was effective in improving ITN use, IPTp uptake, and haematocrit levels. It is, therefore, recommended for the modules to be adopted and incorporated into the routine antenatal care programmes in health centres with predominantly Hausa speaking clients.

Trial registration: Pan African Clinical Trial Registry, PACTR201610001823405. Registered 26 October 2016, www.pactr.org.

## Background

Malaria infection remains endemic in many countries including Nigeria [1], which contributed the largest percentage of cases (27%) to its global incidence in the year 2016 [2]. Complications like anaemia [3, 4], abortion, [5, 6], stillbirth [7, 8], low birth weight [9, 10], and preterm delivery [7] could arise from malaria infection during pregnancy. The World Health Organization (WHO) recommends that all pregnant women living in malaria-endemic regions of sub-Saharan Africa always sleep under an insecticide-treated net (ITN), and take monthly doses of intermittent preventive treatment in pregnancy (IPTp) with sulfadoxine-pyrimethamine (SP), starting from the second trimester of pregnancy [11]. Systematic reviews of trials have also shown that sleeping under an ITN [12, 13] and taking IPTp [14, 15] greatly decrease the risk of malaria infection and its complications during pregnancy.

In a study conducted from 2007 to 2008, 60% of randomly selected pregnant women in a tertiary health centre in Maiduguri, Nigeria, tested positive for malaria parasite at the time of booking for antenatal care [16], and 33.9% of them had placental parasitaemia at the time of delivery [17]. In 2014, the prevalence of malaria parasitaemia among antenatal care attendees in the same centre was 44.5% [18], and 48.1% in a secondary health centre in the same city [19]. Even the adverse events which could occur as complications of malaria in pregnancy, have been highly prevalent in Maiduguri. A four-year retrospective study (1995 to 1998) at a tertiary centre in Maiduguri revealed a neonatal mortality rate of 349 per 1,000 live births; and 62.1% of these deaths were among pre-term infants [20]. In 2012, out of 718 women admitted to the gynaecology ward in the three main public hospitals in Maiduguri, 17.5% had a previous history of spontaneous abortion, 23.1% of which were attributed to infection/malaria and typhoid [6]. Over a four-year period (2001 to 2004) in a tertiary hospital in Maiduguri, there were 23 stillbirths per 1,000 live births, out of 7,996 deliveries [21]. Also in the same centre in 2009, out of 854 pregnant women followed up to delivery, 16.9% had low birth weight babies [22].

Despite the high prevalence of malaria infection recorded among pregnant women accessing health facilities in Maiduguri, the National Demographic and Health Survey of 2014 revealed that in Borno state, only 13.8% of pregnant women aged 15–49 years had slept under an ITN on the night before the survey, while only 13.9% had received any single dose of IPTp [23]. Following the Boko Haram insurgency, Maiduguri now has internally-displaced persons (IDPs) from various local government areas of Borno and some neighbouring states [24]. Of all the 36 states in Nigeria, Borno state has the highest number of IDPs, numbering 672,714, with Maiduguri hosting 432,785, as at 2017 [25]. This is important, as being internally-displaced has been associated with many health problems [26].

Notwithstanding the high prevalence of malaria among pregnant women in the region, the serious associated complications, and the low compliance with prescribed preventive measures, no interventions have been specifically developed for antenatal care attendees to help boost their level of compliance with these preventive practices, and by extension, reduce the incidence of malaria infection and its adverse consequences. A health education intervention on malaria, based on the Protection-Motivation Theory (PMT) was effective in improving both self-efficacy and preventive practices [27], which is a pointer to the important role health theories are likely to play in achieving a health behaviour change. However, the PMT fails to identify other environmental and cognitive factors that can affect attitude change [28].

The Information-Motivation-Behavioural skills (IMB) theory on the other hand, conceptualizes the psychological determinants of performing health behaviours that have an impact on health status. It was first developed to help guide the development of an intervention for HIV preventive behaviours among students. It has three components, which are information about the health behaviours, motivation to carry out such behaviours, and the requisite skills for performing such behaviours [29]. These appear relevant, as higher levels of knowledge on malaria prevention alone, did not always translate to increased use of bed nets [30]. The IMB model asserts that information on malaria prevention, motivation, and behavioural skills are fundamental determinants of malaria preventive behaviour. Another advantage of the IMB model is that it can be culturally-tailored to suit local needs.

The IMB model has proven valid in explaining a number of health behaviours. An IMB assessment of rational drug use behaviour among second-level hospital outpatients in Anhui, China, showed that this behaviour was predicted by greater knowledge (β = 0.25, *p* < 0.001), more motivation (β = 0.42, *p* < 0.001), and better behavioural skills (β = 0.16, *p* < 0.001). In addition, there were significant indirect effects of greater knowledge (β = 0.02, *p* < 0.05) and more motivation (β = 0.07, p < 0.05) on behaviour [31]. In community cultural centres in Isfahan city, Iran, logistic regression results demonstrated that information (OR = 1.071, *p* < 0.001), motivation (OR = 0.978, *p* = 0.045) and behaviour skills (OR = 1.033, *p* = 0.001) predicted breast self- examination among women aged 20–69 years [32]. For insecticide-treated net use among pregnant women in a health facility in Maiduguri, Nigeria, Information and motivation were significantly related to behaviour (*r* = 0.22, *p* < 0.001 and *r* = 0.11, *p* = 0.033 respectively), but behavioural skills did not significantly relate to behaviour (*r* = 0.03, *p* = 0.278) [33]. With regards to consistent condom use (CCU) among unmarried female migrants in China, behavioural skills had a positive effect (β = 0.344, p < 0.001), motivation had no direct influence, but indirectly, through behavioural skills (β = 0.800, p < 0.001), while information had no influence at all on CCU [34]. Similar findings were reported with CCU among transgender women in Shenyang, China, were HIV-preventive motivation (β = 0.823, *p* < 0.001) and behavioural skills (β = 0.979, *p* = 0.004) were related to more frequent condom use, whereas HIV knowledge was unrelated to condom use (β = 0.052, *p* = 0.540) [35].

In addition to its relevance in explaining health behaviours, numerous interventions guided by the IMB model were successful in achieving positive behavioural changes. A health education intervention based on the IMB model not only had large effects on the information (partial ἠ^2^ = 0.32) and motivation constructs (partial ἠ^2^ = 0.17), but also revealed a strong relationship between constructs of the model (information, motivation and behavioural skills) and adherence to medical recommendations and health advices among coronary artery bypass graft (CABG) surgery patients in Tehran, Iran [36]. A randomized controlled trial for HIV prevention among truck drivers in India in which participants were classified into two groups (the intervention group which underwent an IMB-based intervention workshop; and a control group that received an information-only workshop) revealed evidence of effectiveness in improving attitude and increasing HIV preventive behaviour among the intervention group [37]. The IMB model has also been used to predict not only behavioural change, but also the outcome of behavioural change. An intervention study among Puerto Rican Type II diabetes mellitus patients on exercise and dietary behaviour, revealed that for diet, information and motivation predicted behavioural skills, and behavioural skills predicted behaviour, but only behaviour predicted HbA1C control. For exercise, attitude predicted behavioural skills and behavioural skills predicted behaviour; but behaviour in this case did not predict HbA1C [38].

Most of the prior studies with the IMB model have centred on improving preventive health behaviours, which ITN use and IPTp are not exceptions. Also, since the IMB model was useful in explaining ITN use among pregnant women [33], an intervention guided by it, is likely to be successful in boosting their compliance with the recommended malaria preventive measures. This study aimed to determine the effects of a health education intervention based on the information-motivation-behavioural skills theory on malaria knowledge, motivation and behavioural skills, as well as malaria preventive practices and pregnancy outcomes. The intervention was successful in improving the primary outcomes, which are psychological constructs (malaria knowledge, motivation and behavioural skills) as already presented elsewhere [39]. This paper therefore, focuses on the secondary outcomes, which are behavioural (ITN use and IPTp uptake) and clinical factors (haematocrit, malaria infection, and pregnancy outcomes).

## Methods

### Study area

The study area was Maiduguri, the Borno state capital which is located in North-eastern Nigeria. Borno state lies between latitudes 10° 30′ and 13° 50′ north and longitudes 11.00° and 13° 45′ east, with a total land area of 69,435 km^2.^ The climate varies according to the time of the year, with temperatures ranging from 25 °C to 44 °C, and an average annual rainfall of 613 mm [40]. Its population is reported to be 540,016 consisting of 282,409 males and 257,607 females [41]. The study location which was the State Specialist Hospital, is one of the three secondary-level hospitals in Maiduguri, Bono State. It was chosen because it happens to be the biggest of them in terms of size, man-power, and patient load. Its central location in Maiduguri also makes it the most geographically accessible, to the majority of Maiduguri populace. The ante-natal care clinic is run as a unit under the Department of Obstetrics and Gynaecology, and is run from Mondays to Fridays. Mondays are reserved for antenatal care bookings, while follow-up visits are conducted on the other days. Each antenatal booking clinic session has an average load of approximately a hundred clients. Antenatal booking at the hospital’s clinic is open to all pregnant women, irrespective of their gestational ages, and with no requirement for referral letters from other centres. Routine antenatal health education is given by the midwives at the waiting hall, before antenatal care consultations start. These talks given are not structured, and cut across issues of hygiene, proper nutrition, breastfeeding, malaria prevention and any health issue deemed relevant by the staff.

### Study design and study population

The study was a double-blind parallel-group randomized controlled trial guided by the CONSORT Statement [42]. Participants were randomly selected from the ante-natal care attendees, and then randomly assigned to either the intervention or control group (with an equal number of participants in each group). The intervention group received the IMB-based health education intervention on malaria, while the control group received health education on breastfeeding. The study population was pregnant women attending the State Specialist Hospital, Maiduguri, for their ante-natal care.

Those who do not understand the Hausa language were excluded because the intervention was prepared and delivered in the Hausa language. Also excluded were participants who had a history of medical conditions which could interfere with the study outcomes like hypertension, pre-eclampsia or eclampsia [43–45], diabetes [46, 47] and a history of per vaginal bleeding in current pregnancy [48]. Substituting the results of post-intervention ITN use from a previous study (p_1_ = 0.38, q_1_ = 0.62, p_2_ = 0.21, q_2_ = 0.79) [49] into the sample size formula for randomized controlled trials with binary outcomes [50], with a minimum difference of 10% to be detected, at 95% level of significance and power of 80%, gave a minimum required sample size of 346. This shows that the total number of participants–372–for the primary outcome variables [39] is also adequate for the secondary variables.

### Randomization

Random assignment of the participants to either the intervention or control group, was done on the same day the participants were selected into the study. It was however performed after baseline data had been collected.

### Sequence generation

The permuted block randomization technique was used. Permuted blocks of four (each containing two interventions and two controls), containing all the six possible combinations (AABB, BBAA, ABBA, BAAB, ABAB, BABA) were generated using the random function in Microsoft Excel 2013.

### Allocation concealment mechanism

The sequences generated were then placed inside opaque envelopes and sealed by the medical records’ staff who generated them. The envelopes were also serially numbered from the outside, to guide those doing the allocation.

### Implementation

The sequence generation was performed by a trained staff of the hospital’s Medical Records Department, who was not part of any of the other research processes. Two other staff of the antenatal clinic worked independently to assign groups to the participants. The first staff serially handed over the envelopes to the participants without opening, then directed them to the second staff. The second staff then opened the envelopes, informed them of the day to come for their health education session (based on the group to which they belonged), and then documented their respective groups on a sheet provided. Each participant was then given a hand card, which carried their bio-data and serial numbers. The hand card also contained a short note indicating that they had been enrolled into a follow-up study and requesting of the attending physician or nurse to kindly complete the post-natal findings as indicated on the card.

### Blinding

The study was double-blinded, as participants and assessors (enumerators) were blinded to the assigned interventions. The list of assignments was then kept confidential by the staff who documented the participants’ allocations. This staff was only aware of the group coding (A and B), but unaware of the interventions assigned to them. To ensure blinding of the participants, the groups they belonged to, were not indicated on their cards. The subsequent clinic appointment dates for the two groups were set on different days, to minimize contamination. The enumerators were blinded, as they were not involved in any of the processes of group allocation, or delivery of the intervention.

### Development of the health educational intervention modules

For the malaria health education intervention, the information sources for developing the modules were: the National Guidelines and Strategies for Malaria Prevention and Control During Pregnancy, a publication of the Nigerian Federal Ministry of Health [50]; WHO published materials [11, 51]; a publication of the Global Health Learning Centre on Malaria in Pregnancy [52]; and other publications from studies carried out in Nigeria. It was developed based on the Information-Motivation-Behavioural Skills theory [29], using local scenarios for demonstration purposes. The breastfeeding modules were developed from the following sources: National Policy on Infant and Young Child Feeding in Nigeria [53]; Lactation Management Self-Study Modules, Level I [54] and other studies conducted in Nigeria on breastfeeding practices. The items extracted from these sources were compiled to follow a similar pattern with that for the malaria health education intervention. Being prospective mothers, health education on breastfeeding was relevant to them, and as such, had the potentials of serving as a potent placebo intervention.

### Structure of the malaria health educational intervention modules

The intervention consisted of four modules, covering each of the constructs of the IMB model as presented in Table 1. The first and second modules covered the information construct, the third module covered the motivation construct, while the fourth module covered the behavioural skills construct.

**Table 1:** Tabular illustration of the malaria intervention modules by construct and contents

Theory Construct	Module and Strategy	Contents	Estimated time
Information	Modules 1 and Module 2 (Lectures)	Transmission; Clinical features; Complications; Prevention measures	30 min and 30 min
Motivation	Module 3 (Interactive discussion; Brainstorming)	Participants’ experiences and those of other pregnant women	1 h 30 min
Behavioural skills	Module 4 (Lectures; Videos; Demonstrations)	Evaluation of self-efficacy for taking IPTp-SP and using ITN; Goal setting	1 h 30 min

The first module was titled ‘Understanding Malaria in Pregnancy’. This module gave a general discussion on malaria in pregnancy, at the end of which the participants were expected to be able to know what causes malaria; its mode of transmission; its signs and symptoms; its complications during pregnancy; and how it can be prevented. This section was delivered basically through lectures. The second module was titled, ‘The Main Preventive Measures for Malaria in Pregnancy’ which introduced the two most important preventive measures for malaria during pregnancy (TN use and IPTp uptake). This module discussed the importance and efficacy of these preventive measures, as well as success stories of these measures as reported in prior studies. Participants were expected to be able to highlight the protective importance of IPTp and ITN at the end of this section. This section was also delivered through lectures.

The third module was titled, ‘Motivation for Malaria Prevention during Pregnancy’, and it dwelt on motivating the participants to adopt these protective measures. This module was designed to guide an interactive session, for misconceptions and other possible deterrent factors to be addressed interactively. The facilitator together with the participants was to brainstorm and proffer possible ways of overcoming those deterrent factors. At the end of this session, participants were expected to be convinced of the efficacy and safety of these preventive measures, and also motivated to carry them out. The fourth module titled, ‘Insecticide Treated Net and Fansidar’, focused on empowering the participants. They were to be practically shown how to hang and care for their insecticidal nets, and how to look out for defects in their nets, and how to repair them. They were also taught about the timings, indications and contra-indications for IPTp, and also educated on how to take their drugs. Their level of self-efficacy was to be assessed at a group level, and collectively, goals were set to achieve better adherence to these malaria preventive measures. There were evaluation questions at the end of Modules 1, 2 and 4.

### Structure of the breastfeeding health educational intervention modules

The placebo intervention was made to comprise of three modules, each covering a construct of the IMB model. As presented in Table 2, the first module covered the information construct, the second module covered the motivation construct, and the third module covered the behavioural skills construct. The first module titled, ‘An Overview of Breastfeeding’ had two sections: the first section was on the benefits of breastfeeding and the consequences of not breastfeeding; while the second section discussed the practices to avoid during breastfeeding, alternatives to breastfeeding, and challenges associated with breastfeeding, such as sore nipples, low milk supply and breast engorgement. The second module titled, ‘Motivation on Breastfeeding’ contained a list of misconceptions and other deterrent factors for exclusive breastfeeding as identified in previous studies. These were to be discussed and brainstormed by the participants under the facilitator’s guidance to come up with solutions. The third module titled, ‘Practical Session’ was for breastfeeding procedures to be demonstrated practically using models, as well as video demonstrations to be shown to the participants.

**Table 2:** Tabular illustration of the breastfeeding intervention modules by construct and contents

Theory Construct	Module and Strategy	Contents	Estimated time
Information	Module 1 (Lectures)	Benefits of breastfeeding, Consequences of not breast feeding Problems associated with breastfeeding	30 min 1 h
Motivation	Module 2 (Interactive discussion; Brainstorming)	Participants’ experiences and those of other pregnant women	1 h 30 min
Behavioural skills	Module 3 (Lectures; Videos; Demonstrations)	Practical demonstrations of breastfeeding techniques; expression of breast milk	1 hour

### Quality control of the health educational interventions

This entailed appraising the modules for accuracy, relevance and comprehensibility, as well as giving the facilitator adequate training on the modules.

### Quality control of the module contents

The modules for the intervention group as well as the control group were appraised for their contents and relevance by expert teams. For the malaria health education intervention modules, the team comprised of three public health specialists, an obstetrics and gynaecology specialist, a health educator, and a midwife; while the appraisal team for the modules on breastfeeding comprised of three public health specialists, a health educator, and a paediatric nurse. Both interventions were pre-tested with a sample of twenty-five pregnant women from the same hospital, who were not part of the study. Feedback was then obtained from the experts, as well as the participants, and appropriate modifications and corrections were effected on the modules.

### Training of the module facilitator

One facilitator who was a midwife was chosen to deliver the modules at all the health education sessions for both groups. For each of the two interventions, the facilitator received two sessions of one-on-one training by the researcher. The first session lasted about four hours long, during which the contents of the modules, and how to deliver them within their limits were treated. A revision session was then held again in a similar way after the module had been revised (after the pre-testing).

### Delivery of the malaria IMB-based malaria health educational intervention

The interventions were given a week after baseline data had been collected. As suggested by McMillan, delivery of educational interventions should be in modes that are consistent with what is used in practice [55], and as such, they were given at a group level, and in a single session, with each session having around fifty participants. The module was delivered by the facilitator (a midwife nurse), to eliminate experimenter effects. The facilitator was blinded to the objectives of the study, and delivery of the module was also closely monitored and supervised by the researcher, while giving necessary feed-back to the facilitator to ensure that the module was strictly followed without straying. The facilitator went through all the four modules, while actively engaging the participants. The durations were approximately 30 min, 30 min, 1 h 30 min, and 1 h 30 min, for modules 1, 2, 3 and 4 respectively, with breaks of about 10 to 15 min between modules. Each participant was given the chance to demonstrate what she had learnt, and corrective feed-back was given.

### Delivery of the breastfeeding health education intervention

The health education modules on breastfeeding were delivered by the same midwife who gave the malaria health education. The total duration of the session was also around four hours, and the delivery was of similar methodology to that of the malaria health education modules, comprising of lectures with power-point presentations, videos, discussions, and practical sessions. A paediatric nurse helped out with the demonstrations using models. The delivery was also strictly monitored to ensure strict adherence to the module and its contents, without straying.

### Follow-up of participants

After collecting baseline data, randomization, and delivery of the intervention, participants were then followed up. Follow-up data were collected using the same questionnaire, at 2-months post-intervention and 4-months post-intervention. Participants were also requested to always carry their hand cards with them each time they visited the hospital, especially when they were going for delivery, or in the case of home delivery, to present to the hospital immediately. Data on babies’ birth weights and pregnancy outcomes were filled on the hand card by the attending physician or nurse, which participants submitted back after their deliveries. For each visit, compensation for transport fare was given to each participant.

### Variables

The independent variable in this study was group, whereas the dependent variables were reported ITN use, reported IPTp uptake, reported malaria diagnosis, haematocrit, pregnancy outcome, and babies’ birth weights. Other variables which were considered potential confounding factors in this study, included ethnicity, family type, type of residence, educational status, occupational status, income level, age at first marriage, gravidity; parity, period of amenorrhoea, history of a previous preterm delivery, history of a previous miscarriage, time, and group-time interaction.

Group, was defined as the study arm a participant belonged to, which was either the intervention or control group. Time meant the three time points, from baseline to 2-months post-intervention, to 4-months post-intervention, while group-time interaction entailed the effect of the intervention with regards to time. Reported malaria diagnosis was said to be positive if a participant reported that she had been diagnosed with malaria in a health centre after having had a blood test done. A monogamous family was defined as one with one husband and one wife, while a polygamous family was defined as one in which the husband had more than one wife [56]. An internally displaced person (IDP) was defined as a Nigerian who had been displaced from her town of residence, but not outside the country, as a result of armed conflict situation [57]. The Nigerian minimum wage of N18,000 was used to categorize monthly income. Live birth was defined as one in which there was any sign of life after expulsion from the mother, irrespective of the gestational age [58], while a stillbirth was defined as a baby born after 28 weeks of gestation with no signs of life [59]. A baby was defined as low birth weight if its birth weight was below 2.5 kg [60].

### Instruments and data collection methods

The instruments used for data collection in this study were the questionnaire and study hand card. Due to low literacy rates, the questionnaires were administered as face-to-face interviews in Hausa language, by trained enumerators. The questionnaire had four sections: socio-demographic characteristics; obstetric and gynaecological history; preventive measures; malaria infection and haematocrit. The section on preventive measures asked about how frequently they slept under an ITN, and whether or not they had taken any IPTp, and if yes, the number of doses. Frequency of ITN use was categorized into levels, as Never, Seldom (1–2 times a week), Sometimes (3–4 times a week), Often (5–6 times a week) and Almost always. These levels were scored: 1, 2, 3, 4 and 5, respectively. Their haematocrit measurements were obtained from their ante-natal cards, as it is routinely done in the ante-natal clinic.

The study hand cards were designed by the researchers to collect information about post-pregnancy events. They were given to the participants immediately after recruitment, and were to be submitted to the researcher at the end of the pregnancy. It contained the participants’ serial numbers, basic bio-data, and a short appeal to the attending physician or nurse, informing them that the patient had been enrolled into a follow-up study, and his/her help was required to kindly complete the blanks provided on the card. The items it contained were: outcome of pregnancy (live birth, stillbirth or miscarriage), place of delivery (hospital/health centre or home) and baby’s birth weight. For those who gave birth at home, only information on pregnancy outcome and place of delivery were collected when they came to submit the study cards.

### Statistical analyses

Data collected from the questionnaires and study hand cards (Additional file 1) were entered into the IBM Statistical Package for Social Sciences (SPSS) version 22, followed by data cleaning. The data set for this study are available as supplementary material. Histogram and absolute values of skewness and kurtosis of not more than 2 and 7 respectively, were used to check for normality of the haematocrit and birth weights [61]. The outcome showed that there was no substantial non-normality in these continuous variables, and as such the data could be treated as normally distributed. Mean and standard deviation were used to summarize continuous variables, while the categorical variables were summarized as frequency and percentage. Pearson’s Chi-squared test was used to compare the baseline and follow-up categorical variables of the two groups. Fisher’s Exact test was performed for variables where any cell had less than five expected observations. Independent t-test was performed on the haematocrit and birth weights to determine the between-group differences in these variables. The mixed design repeated measures Analysis of Variance (ANOVA) was performed to determine the between and within-group differences, as well as the interaction between group and time on mean haematocrit levels.

The intention-to-treat (ITT) analysis was performed after replacing missing values [62], for which the multiple imputations method was used. This method was chosen since some correlations were expected between the missing observations, such that non-missing values on one outcome can inform the imputation of missing values on another outcome [63]. An exploration of the missing data showed that 11.09% and 13.96% of the data of the intervention and control groups were missing respectively. The Little’s Missing Completely at Random (MCAR) test was not significant (*χ*^*2*^ = 140, *df* = 137, *p* = 0.400) for both groups, making the multiple imputations method suitable [64]. The baseline variables were used only for prediction, while the outcome variables were imputed and used for prediction as well. The output of the multiple imputations analysis generated five different data sets which is the default in SPSS, each with missing values replaced. These were then pooled into a single data set for subsequent analyses.

The Generalized Linear Mixed Models (GLMM) analysis was performed to determine the overall effectiveness of the intervention [65]. Twelve potential confounding factors (ethnicity; family type; type of residence, educational status, occupational status, income level; age at first marriage; gravidity; parity level; period of amenorrhoea; history of a previous preterm delivery, and history of a previous miscarriage) plus the baseline data for the outcome variable in question were included in the GLMM analysis. The combination of these variables gave the best model, with the lowest Akaike Corrected Information Criterion (ACIC) and Bayesian Information Criterion (BIC). The small intervention groups were entered as the third cluster level, after the identifier (serial number) and intervention group. For sensitivity analysis, the GLMM analysis was repeated without the replacements (per-protocol), to determine the effect of drop out and non-response on the findings of the study. For all analyses, Confidence Interval (CI) of 95% was used, and level of significance was set at 0.05.

## Results

**Fig. 1:**
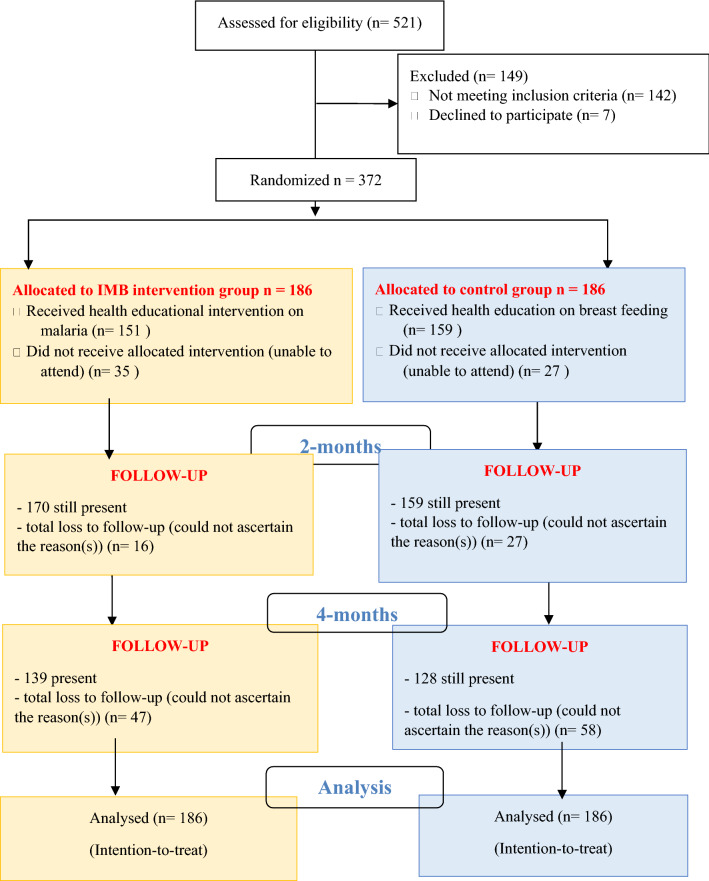
Consort Flow Chart of Intervention and Control Groups

The stages through which participants were finally selected into the study are presented in Fig. 1. Study participants were recruited from eight antenatal booking clinic sessions from 30 January 2017 to 13 March 2017 and the last data on pregnancy outcomes was obtained on 21 September 2017, after which no more participant had returned. Three hundred and seventy-two pregnant women were finally selected to participate in the study, with 186 in the intervention group and 186 in the control group. Most of the participants in the intervention (81.2%) and control groups (85.5%), had attended their respective health education sessions. At 4-months post-intervention, 25.3% and 31.2% from the intervention and control groups respectively, had dropped out of the study.

### Participants’ baseline characteristics

Table 3 compares the baseline socio-demographic and obstetric and gynaecological characteristics of the intervention and control groups. The intervention group had a higher proportion of IDPs compared to the control group. There was no significant difference between the groups in terms of their age groups of first marriage, gravidity, parity, gestational age at the time of antenatal booking, history of a previous preterm delivery or miscarriage.

**Table 3:** Baseline comparison of socio-demographic and obstetric and gynaecological characteristics between intervention and control groups

Socio-demographic factor	Groups			
	Intervention Freq. (%) (n = 186)		Control Freq. (%) (n = 186)	
Age (years)				
Mean ( SD)	26.1	(5.8)	26.9	(5.9)
(Range)		(15–40)		(17–45)
Ethnicity				
Kanuri	73	(37.7)	63	(33.9)
Hausa	30	(16.1)	28	(15.1)
Babur	13	(7.0)	18	(9.7)
Shuwa	6	(3.2)	12	(6.4)
Marghi	14	(7.5)	12	(6.4)
Fulani	21	(11.3)	17	(9.1)
Others	32	(17.2)	36	(19.4)
Family type				
Monogamous	143	(76.9)	144	(77.4)
Polygamous	41	(22.0)	42	(22.6)
Widowed	2	(1.1)	0	(0.0)
Educational status				
None	82	(44.1)	71	(38.2)
Primary	32	(17.2)	32	(17.2)
Secondary	54	(29.0)	54	(29.0)
Tertiary	18	(9.7)	29	(15.6)
Employment status				
None	104	(55.9)	101	(54.3)
Self-employed	56	(30.1)	65	(34.9)
Government	9	(4.9)	9	(4.9)
Private	14	(7.5)	10	(5.4)
Student	3	(1.6)	1	(0.5)
Income				
None	106	(57.0)	102	(54.8)
< 18,000	65	(34.9)	72	(38.7)
≥ 18,000	15	(8.1)	12	(6.5)
Type of Residence				
Permanent resident	127	(68.3)	147	(79.0)
IDP	59	(31.7)	39	(21.0)
Age at first marriage				
≤ 15	41	(22.0)	35	(18.8)
16–19	105	(56.5)	100	(53.8)
20–24	32	(17.2)	40	(21.5)
≥ 25	8	(4.3)	11	(5.9)
Gravidity				
Primigravida	24	(12.9)	24	(12.9)
Multigravida	96	(51.6)	96	(51.6)
Grandmultigravida	66	(35.5)	66	(35.5)
Parity				
Nullipara	28	(15.1)	25	(13.4)
Primi-para	39	(21.0)	40	(21.5)
Multipara	77	(41.4)	75	(40.3)
Grandmultipara	42	(22.6)	46	(24.7)
Gestational age at antenatal booking				
4 months	49	(26.3)	65	(34.9)
5 months	137	(73.7)	121	(65.1)
History of pre-term delivery				
Yes	37	(19.9)	36	(19.4)
No	149	(80.1)	150	(80.6)
History of miscarriage				
Yes	49	(26.3)	53	(28.5)
No	137	(73.7)	133	(71.3)

### Participants’ reported baseline preventive practices, reported malaria diagnosis, and haematocrit

Most of the respondents (89.24%) reported ever sleeping under any type of mosquito net, but only 43.0% reported ever sleeping under an insecticide-treated net during their current pregnancy. Less than a half (42.7%) had received their first dose of IPTp. Three hundred and twenty three respondents (86.82%) had their haematocrit results at baseline, with values ranging from 20.0% to 43.0%, and a mean (SD) haematocrit of 32.8 (3.9) per cent. Table 5 shows that both groups did not differ significantly in their reported use of ITN, IPTp uptake, reported malaria diagnosis, and haematocrit at baseline.

### Follow-up comparison of reported malaria preventive practices and maternal clinical variables between the groups

As shown in Table 4, reported ITN use and IPTp uptake were higher among participants of the intervention group compared to the control group at two-months and four-months post-intervention. Reported malaria diagnosis was higher among the control group at both time points, whereas, the intervention group had a higher mean haematocrit level at two-months post-intervention (*t* (251, 250.72) = 2.649, *p* = 0.009) (Table 5).

**Table 4:** Comparison of reported malaria preventive practices between the groups, at baseline, two months, and four months post-intervention

Variable	Group				*χ*^*2*^	*df*	*p*
	Intervention Freq. (%) n = 186		Control Freq. (%) n = 186				
Baseline							
Reported ITN use					7.434	4	0.115
Never	113	(60.8)	99	(53.2)			
Seldom	10	(5.4)	18	(9.7)			
Sometimes	20	(10.7)	16	(8.6)			
Often	26	(14.0)	23	(12.4)			
Almost always	17	(9.1)	30	(16.1)			
2 months							
Reported ITN use					27.974	4	< 0.001
Never	38	(22.4)	40	(25.2)			
Seldom	11	(6.5)	38	(23.9)			
Sometimes	32	(18.8)	32	(20.1)			
Often	51	(30.0)	22	(13.8)			
Almost always	38	(22.4)	27	(17.0)			
4 months							
Reported ITN use					12.905	4	0.012
Never	30	(21.6)	35	(27.3)			
Seldom	8	(5.8)	22	(17.2)			
Sometimes	28	(20.1)	24	(18.8)			
Often	39	(28.1)	22	(17.2)			
Almost always	34	(24.5)	25	(19.5)			
Baseline							
Reported doses of IPTp taken					0.275	1	0.600
Yes	77	(41.4)	82	(44.1)			
No	109	(58.6)	104	(55.9)			
2 months							
Reported doses of IPTp taken					10.609	2	0.005
None	24	(14.1)	40	(25.2)			
One	103	(60.6)	97	(61.0)			
Two	43	(25.3)	22	(13.8)			
4 months							
Reported doses of IPTp taken					26.350	3	< 0.001
None	6	(4.3)	10	(7.8)			
One	20	(14.4)	47	(36.7)			
Two	82	(59.0)	62	(48.4)			
Three	31	(22.3)	9	(7.0)

**Table 5:** Comparison of maternal clinical conditions between the groups, at baseline, two months post-intervention, four months post-intervention

Variable	Group				*χ*^*2*^	*df*	*p*
	Intervention Freq. (%) n = 186		Control Freq. (%) n = 186				
Baseline							
Reported malaria diagnosis					0.650	1	0.420
Yes	49	(26.3)	56	(30.1)			
No	137	(73.7)	130	(69.9)			
2 months							
Reported malaria diagnosis					4.345	1	0.037
Yes	76	(46.1)	90	(57.7)			
No	89	(53.9)	66	(42.3)			
4 months							
Reported malaria diagnosis					4.910	1	0.027
Yes	36	(25.9)	49	(38.6)			
No	103	(74.1)	78	(61.4)			
Baseline							
Haematocrit					1.573	321	0.117^a^
Mean (SD)	33.09	(4.00)	32.42	(3.71)			
2 months							
Haematocrit							
Mean (SD)	31.93	(4.09)	30.63	(3.70)			
End of pregnancy							
Birth outcome					-	-	0.623
Live birth	140	(97.9)	137	(99.3)			
Stillbirth	3	(2.1)	1	(0.7)			
End of pregnancy							
Birth weight category					1.780	1	0.182
Normal birth weight	98	(77.8)	88	(70.4)			
Low birth weight	28	(22.2)	37	(29.6)			

( ^a^) – *p* for t-test

### Comparison of pregnancy outcomes and birth weights between the groups

Out of the 281 participants from whom post-delivery information was obtained, 257 (91.5%) had hospital deliveries. Also, while four participants had reported having a stillbirth, none had reported having a miscarriage. The birth weights of 251 babies were retrieved, which ranged from 1.20 to 4.30 kg, with mean (SD) weight of 2.75 (0.60) kg. As presented in Table 5, there was no significant difference between the birth weights of the two groups (*t* = 1.894, *df* = 249, *p* = 0.059). There was also no significant difference in the occurrence of stillbirths between groups (*p* = 0.623). Also, 22.2% and 29.6% respectively, of participants in the intervention and control groups had babies with a low birth weight, with no significant differences in the proportion of low birth weight babies between the groups (*χ*^2^ = 1.780, *df* = 1, *p* = 0.182).

### Effect of the intervention on the outcome variables

The effects of group, time, and group-time interaction on reported ITN use and IPTp uptake, as well as haematocrit level, were all significant. For the frequency of reported malaria diagnosis, there was only a significant effect for time. There was no significant effect for group on babies’ birth weights (Additional file 2).

For the interaction plots between group and time (Additional file 3), there was an increase in the reported of ITN use among both the intervention and control groups from baseline to two months post-intervention, followed by a mild drop for the intervention group from two months post-intervention to four months post-intervention. For the control group however, there was a further increase from two months post-intervention to four months post-intervention. There was a progressive increase in the number of IPTp doses reported to have been taken, from baseline to four months post-intervention for both groups. Reported ITN use and IPTp uptake however, persistently remained higher, for the intervention group compared to the control group. There was also a significant drop in haematocrit levels for both groups.

### The magnitude of the intervention effect

Table 6 illustrates the magnitude of the intervention effect in this study. A participant in the intervention group reportedly slept under an ITN by an additional 0.32 more levels, and took 0.37 more doses of IPTp than a participant in the control group. A participant in the intervention group also achieved a haematocrit level of 0.80% above a participant in the control group. There were no significant differences in reported malaria diagnosis, as well as babies’ birth weights between the two groups. For the sensitivity analysis, a comparison of the fixed coefficients for group, with, and without replacement of missing values showed that the effect of the intervention on IPT uptake and haematocrit were negatively affected by drop out, while drop out had a positive impact on the other variables (Additional file 2).

**Table 6:** Fixed coefficients of the outcome variables

Variable	Coefficient	Sig	95% C.I	
			Lower	Upper
Reported ITN use				
Intervention	0.32	0.018	0.06	0.59
Control	0			
Reported IPTp uptake				
Intervention	0.37	< 0.001	0.26	0.47
Control	0			
Reported malaria diagnosis				
Intervention	− 0.18	0.213	− 0.450	0.100
Control	0			
Haematocrit				
Intervention	0.80	< 0.001	0.53	1.07
Control	0			
Birth weight				
Intervention	0.08	0.236	− 0.05	0.22
Control	0			

Adjustment made for 12 potential confounding variables with missing data replaced

## Discussion

A single health education session on malaria prevention during pregnancy (based on the IMB model) was implemented to determine its effectiveness in improving malaria preventive practices and pregnancy outcomes among pregnant women. At the end of the study, reported ITN use, reported IPTp uptake, and haematocrit levels, were significantly higher among those in the intervention group compared to the control group. However, there was no significant difference in the frequency of reported malaria diagnosis, and babies’ birth weights between the two groups. The proportion of participants who completed the study (71.8%), was lower than that for a previous prospective study conducted among pregnant women in Maiduguri (83.3%) by Bako et al*.* [16], which was probably so because the previous study was conducted in a tertiary hospital, and as such, more likely to have had more compliant clients. The aftermath of the Boko Haram insurgency which has been characterized by massive displacements and huge socio-economic loses to the region [24] could also have contributed to some of the attrition in this study. The participants also showed a very poor knowledge of their last menstrual periods, which could be explained by the low level of education and/or poor attitudes towards the dates, as even many who were educated up to tertiary levels did not know theirs.

According to the IMB model, the first constructs, information and motivation, act directly or indirectly through behavioural skills to influence behaviour, which in turn is proposed to influence health status. The findings of this study give further credence to the IMB model, as it can be seen that the effect size of the intervention progressively waned across the model, with the biggest effect size being on the most proximal constructs (malaria knowledge and motivation), followed by behavioural skills [39], and then the health behaviours (reported ITN use and reported IPTp uptake), and lastly health status (the clinical outcomes). As the intervention itself had only components of the IMB, it is not unexpected that the greatest effects of the intervention were on these constructs. The results in this paper and that of the previous analysis [39] also points to the potential role of psychological constructs in determining health status.

The results of the behavioural outcomes could be interpreted at a group-level to mean that for 32% of the participants, an individual in the intervention group is expected to sleep under an ITN, a level (at least one day or at most three days) more frequently; while for 37% of the participants, an individual in the intervention group is expected to take one more dose of IPTp compared to an individual in the control group. Although some previous interventions had also been effective in increasing malaria preventive practices among the intervention groups [27, 49, 66, 67], the intensity of such interventions in terms of duration, had been contrastingly very high (as some lasted for up to a month) when compared to the single session four-hour intervention in this study.

Seasonal variation in malaria prevalence has been reported in Maiduguri, with the highest prevalence in September, followed by June, and the least prevalence in March [68]. Seasonal variation has also been reported in the mosquito population and mosquito biting habit in other parts of Nigeria [69, 70]. Considering that the selection of participants as well as baseline data collection was concluded in March, the increase in reported ITN use with time, could partly be explained by the increase in mosquito presence as the study went on. However, the effect of the intervention is likely to have led to the higher ITN use among the participants in the intervention group.

Around a third of the respondents (30.5%) reported that they had never been diagnosed of malaria during their current pregnancies, 47.6% reported having one episode, 18.7% had two, while 3.2% had had malaria up to three times during their pregnancies. These findings appear very similar to findings among women from selected communities in Badagry, Nigeria, where 36.0% reported having had malaria only once, 18.0% twice, 6% thrice, while 36% had never had malaria during their pregnancies [71]. Even though both groups showed a similar trend of initial increase, followed by a decrease in the incidence malaria diagnosis, it had consistently been higher among the control group since after baseline. A previous study had reported that IPTp was not effective in preventing incident cases of malaria, but rather decreasing the prevalence of those already infected [72]. As such, a higher rate of progression from asymptomatic to symptomatic malaria should be expected in the control group, since the intervention group had a higher IPTp uptake, even though not statistically significant.

Haematocrit measurements were obtained for only two time points (baseline and first follow-up). Both groups had experienced a significant decline in their mean haematocrit levels, from baseline to the time of their first follow-up visits. This decline in haematocrit with advancing pregnancy is consistent with earlier findings, and has been attributed to haemodilution [73, 74]. However, while there were no significant differences between the groups at baseline, the mean haematocrit level for the intervention group was higher than that for the control group at their first follow-up visit.

The incidence of low birth weight was higher than previous findings of 7.8% and 16.9% in hospital-based cohort studies in Maiduguri [16, 22]. The overall prevalence of low birth weight in Nigeria was also lower than the findings of this study (7.3%) [72]. Lower socioeconomic status [75–78] has been associated with the occurrence of low birth weight, which could have probably been the reason for the higher incidence of low birth weight in this study. This presumed lower social status of the participants could be inferred from the hospital type (secondary versus tertiary level), and the presence of IDPs among them. Malaria infection during pregnancy has been associated with low birth weight [10, 79], and as such, since the intervention was not significant in reducing the incidence of malaria, no significant effect would, therefore, be anticipated in babies’ birth weights.

The stillbirth rate of 14/1000 deliveries, in this study, was slightly lower than findings from a four-year study at a tertiary hospital in Maiduguri where 22 stillbirths were reported, per 1,000 deliveries. Even in a tertiary hospital in eastern Nigeria, 18.0% (180 per 1,000) of the deliveries had ended in a stillbirth [80]. A likely explanation for the low stillbirth rates and zero miscarriage in this study, could have been a demoralization on the part of those who had had those experiences, which made them drop out of the study, leading to loss of subsequent information.

One of the strengths of the study was the adequate number of participants, as this was higher than the minimum required sample size calculated. Among other strengths of the study were the random allocation of participants to groups, the placebo intervention given to the control group on a relevant topic like breast feeding, blinding of the participants and assessors, development of a standard module in the target language, and having had all sessions for both groups delivered by the same facilitator. The robust generalized-linear mixed models analysis was also likely to allow for visualization of the pure effects of the intervention. There was no significant difference in attrition between the intervention and control group. The baseline characteristics of those who dropped out and those who remained in the study were also the same, suggesting that attrition was unlikely to have affected the randomization process. In addition, the intention to treat and sensitivity analysis ensured that the randomization was preserved, and allowed for assessing the impact of attrition on the results, respectively.

A major limitation of this study was that the dependent variables (except haematocrit and birth weight) were measured based on self-reporting, rendering the results less accurate against if they had been obtained from direct observation [81]. Reported net use for example, suggests that the person has been in the net all through the night, which may not necessarily be so. Outdoor activities at night [82], multiple exits and entries, with lifting of the nets at night [83], and entering the nets late, are factors which could greatly affect the level of protection given by the net. These are however not captured in this study, as such making it impossible to accurately compare the protection received from insecticidal nets by the two groups. Malaria infection, which was also measured based on self-reporting, was also prone to bias from individuals’ characteristics such as the level of infection (clinical or sub-clinical) and also their level of stoicism. Another limitation of the study was the possibility of contamination from information sharing at home or other meeting places. Attrition of some participants means that there is the possibility that the results obtained are not a hundred per cent accurate, since some of the participants’ information were not actually measured, but approximated. With the major ethnic group in Maiduguri being Kanuri, implementation of the intervention module may be limited in certain parts of the city where Hausa language is poorly understood.

## Conclusions

The intervention was effective in improving reported ITN use, reported IPTp uptake, and haematocrit levels. It is thus recommended that the module be adopted and incorporated into the routine ante-natal care programmes in health centres with predominantly Hausa speaking clients. For this, an extra day visit to the antenatal clinic may be necessary, to adequately cover the contents of the intervention modules. Considering that the study area is a resource-low setting, alternative methods could be explored, followed by a cost-effectiveness analysis of these methods to determine the most suitable option. For example, follow-up studies should be conducted to determine the effectiveness of teaching the modules over several clinic visits, say, covering one module at each visit. If effective, this would eliminate the need for an additional clinic visit while still achieving the desired outcome. Future studies should also consider more objective ways of measuring study variables, such as using early ultrasound scanning to measure the gestational age, and microscopy for malaria parasite, rather than relying on self-reporting which may not be accurate. A tracking system for participants should also be implemented, to minimize loss to follow up.

## Supplementary Information

**Additional file 1:** Study Dataset. Spreadsheet of the raw data collected and analyzed in this study.

**Additional file 2: Tables**. Tables of the results of further statistical tests (GLMM and sensitivity analysis).

**Additional file 3: Figures. **Figures of group and time interaction plots for the outcome variables.

## Data Availability

The data set for this study are available as supplementary material.

## Abbreviations

ACIC: Akaike Corrected Information Criterion
BIC: Bayesian Information Criterion
CI: Confidence Interval
GLMM: Generalized Linear Mixed Models
IDP: Internally-displaced person
IMB: Information-motivation-behavioural skills
IOM: International Organization for Migration
IPT: Intermittent preventive treatment in pregnancy
ITT: Intention-to-treat
LMP: Last menstrual period
MCAR: Missing Completely at Random
PMT: Protection-Motivation Theory
SP: Sulfadoxine-pyrimethamine
SPSS: Statistical Package for Social Sciences
WHO: World Health Organization
