# Supplementary material for: Improving malaria preventive practices and pregnancy outcomes through a health education intervention: A randomized controlled trial
Source: Malar J. 2021 Jan 21;20:55. doi: 10.1186/s12936-021-03586-5 (PMC7818731; doi:10.1186/s12936-021-03586-5)
Supplement: Supplementary file 2 — Additional file 2: Tables. Tables of the results of further statistical tests (GLMM and sensitivity analysis). [file 12936_2021_3586_MOESM2_ESM.docx]

**Table 1. Fixed effects of group, time, and group-time interaction on the outcome variables**

| **SOURCE** | **F** | **df1** | **df2** | **Sig.** |
| --- | --- | --- | --- | --- |
| **Reported ITN use** |  |  |  |  |
| Group | 5.90 | 1 | 1062 | 0.015 |
| Time | 56.27 | 2 | 1062 | <0.001 |
| Group*Time | 6.64 | 2 | 1062 | 0.001 |
| **Reported IPTp uptake** |  |  |  |  |
| Group | 35.26 | 1 | 1062 | <0.001 |
| Time | 569.70 | 2 | 1062 | <0.001 |
| Group*Time | 14.27 | 2 | 1062 | <0.001 |
| **Reported malaria diagnosis** |  |  |  |  |
| Group | 3.67 | 1 | 1062 | 0.056 |
| Time | 26.04 | 2 | 1062 | <0.001 |
| Group*Time | 0.21 | 2 | 1062 | 0.808 |
| **Haematocrit** |  |  |  |  |
| Group | 16.64 | 1 | 694 | <0.001 |
| Time | 220.37 | 1 | 694 | <0.001 |
| Group*Time | 17.42 | 1 | 694 | <0.001 |
| **Birth weight** |  |  |  |  |
| Group | 1.41 | 1 | 328 | 0.236 |

Note: Adjustment made for 12 potential confounding variables with missing data replaced

**Table 2. Fixed effects of group, time, and group-time interaction on the outcome variables**

| **SOURCE** | **F** | **df1** | **df2** | | **Sig.** |
| --- | --- | --- | --- | --- | --- |
| **Reported ITN use** |  |  |  |  | |
| Group | 10.08 | 1 | 914 | 0.002 | |
| Time | 47.22 | 2 | 914 | <0.001 | |
| Group*Time | 8.17 | 2 | 914 | <0.001 | |
| **Reported IPTp uptake** |  |  |  |  | |
| Group | 8.80 | 1 | 914 | 0.003 | |
| Time | 410.81 | 2 | 914 | <0.001 | |
| Group*Time | 4.24 | 2 | 914 | 0.015 | |
| **Reported malaria diagnosis** |  |  |  |  | |
| Group | 7.92 | 1 | 905 | 0.005 | |
| Time | 22.46 | 2 | 905 | <0.001 | |
| Group*Time | 1.04 | 2 | 905 | 0.354 | |
| **Haematocrit** |  |  |  |  | |
| Group | 15.68 | 1 | 526 | <0.001 | |
| Time | 162.75 | 1 | 526 | <0.001 | |
| Group*Time | 17.38 | 1 | 526 | <0.001 | |
| **Birth weight** |  |  |  |  | |
| Group | 7.69 | 1 | 207 | 0.006 | |

Note: Adjustment made for 12 potential confounding variables with missing data NOT replaced

**Table 3. Comparison of fixed coefficients for group, with and without imputed missing values**

| **Variable** | **Missing values not imputed** | | **Missing values imputed** | | **Coefficient difference** | **Percentage coefficient difference** |
| --- | --- | --- | --- | --- | --- | --- |
|  | **Coefficient** | **Sig.** | **Coefficient** | **Sig.** |  |  |
| **Reported ITN use** |  |  |  |  |  |  |
| Intervention | 0.50 | 0.003 | 0.32 | 0.018 | 0.18 | 5.63 |
| Control | 0 |  | 0 |  |  |  |
| **Reported IPTp uptake** |  |  |  |  |  |  |
| Intervention | 0.23 | <0.001 | 0.37 | <0.001 | -0.14 | -37.84 |
| Control | 0 |  | 0 |  |  |  |
| **Reported malaria diagnosis** |  |  |  |  |  |  |
| Intervention | -0.432 | 0.014 | -0.18 | 0.213 | - | - |
| Control | 0 |  | 0 |  |  |  |
| **Haematocrit** |  |  |  |  |  |  |
| Intervention | 0.75 | <0.001 | 0.80 | <0.001 | -0.05 | -6.25 |
| Control | 0 |  | 0 |  |  |  |
| **Birth weight** |  |  |  |  |  |  |
| Intervention | 0.22 | 0.006 | 0.08 | 0.236 | - | - |
| Control | 0 |  | 0 |  |  |  |
