# Supplementary material for: Improving malaria preventive practices and pregnancy outcomes through a health education intervention: A randomized controlled trial
Source: Malar J. 2021 Jan 21;20:55. doi: 10.1186/s12936-021-03586-5 (PMC7818731; doi:10.1186/s12936-021-03586-5)
Supplement: Supplementary file 3 — Additional file 3: Figures. Figures of group and time interaction plots for the outcome variables. [file 12936_2021_3586_MOESM3_ESM.docx]

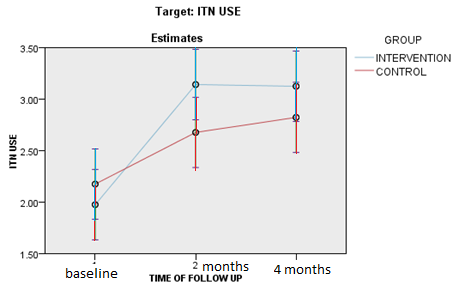


**Figure 1. Interaction Plot between Group and Time for ITN Use.** The vertical axis shows the frequency levels of reported ITN use.


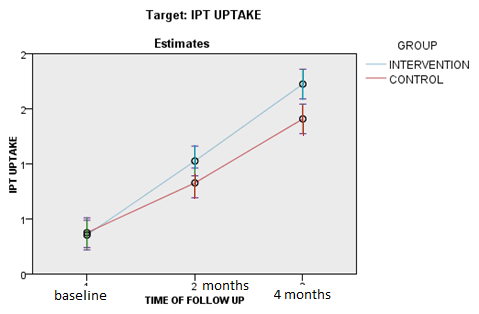


**Figure 2. Interaction Plot between Group and Time for IPTp Use.** The vertical axis illustrates the number of reported IPTp doses taken.

**
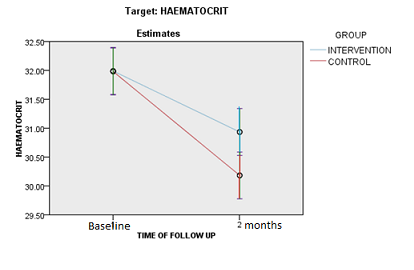
**

**Figure 3. Interaction Plot between Group and Time for Haematocrit.** The vertical axis presents the haematocrit levels in percentage.
